# Suprachoroidal injection in the treatment of diabetic macular edema: mechanisms, clinical advances, and future perspectives

**DOI:** 10.3389/fmed.2026.1797383

**Published:** 2026-05-08

**Authors:** Menghan Chen, Wanzhen Jiao, Tianwei Liu, Bojun Zhao

**Affiliations:** 1 Department of Ophthalmology, Shandong Provincial Hospital Affiliated to Shandong First Medical University, Jinan, Shandong, China; 2 Department of Ophthalmology, Jinan Second People’s Hospital, Jinan, Shandong, China

**Keywords:** diabetic macular edema (DME), intravitreal injections (IVI), microinjector, suprachoroidal space (SCS) injection, triamcinolone acetonide (TA)

## Abstract

Diabetic macular edema (DME) is the leading cause of vision loss in patients with diabetes. Conventional therapies like intravitreal injections (IVI) of anti-vascular endothelial growth factor (VEGF) agents and corticosteroids are often limited by the frequent injections and risks of complications. Suprachoroidal space (SCS) injection has emerged as a novel drug delivery technique. It targets eye precisely, resulting in high drug concentration in the posterior segment while reducing exposure to anterior segment tissues—thereby potentially lowering risks of both cataract formation and elevated intraocular pressure. Clinical data shows that SCS injection of triamcinolone acetonide (TA) can significantly improve vision, reduce both macular edema and treatment frequency in DME patients. In this review, we mainly aim to introduce the advantages and clinical evidence of SCS injection, and emphasize its potential role in optimizing DME management strategy.

## 1 Introduction

DME is a severe microvascular complication of diabetic retinopathy (DR), characterized by increased retinal vascular permeability leading to fluid accumulation in the macula. DME can cause progressive visual impairment and may lead to irreversible vision loss without timely intervention (1, 2). By 2030, the global population affected by DR can reach 130 million (3), with the risk of DME also rising significantly.

Current managements of DME mainly rely on intravitreal injections (IVI) of anti-vascular endothelial growth factor (VEGF) agents, supplemented with IVI of corticosteroids or laser photocoagulation (4). However, these conventional treatments have several drawbacks like frequent injections, risks of ocular complications, and limited therapeutic effects (5–7).

In recent years, SCS injection has become a transformative drug delivery approach. Using a specialized microinjector, this technique enables precise administration of therapeutic agents into the space between the choroid and sclera. It offers distinct advantages, including targeted drug delivery, enhanced bioavailability, prolonged therapeutic effects, and reduced side effects (8).

This review critically examined the pharmacokinetic properties, clinical application and existing challenges of SCS injection in DME patients, with the aim of providing comprehensive evidence to optimize DME treatment.

## 2 Search strategy and selection criteria

To ensure a comprehensive synthesis of the available evidence, we conducted a systematic literature search of the PubMed, Embase, and Web of Science databases for articles published from January 2004 to December 2024. The search strategy employed a combination of the following key terms: “suprachoroidal space,” “suprachoroidal injection,” “diabetic macular edema,” “triamcinolone acetonide,” “corticosteroids,” and “drug delivery.”

Priority was given to peer-reviewed original research and authoritative reviews published in English. To provide foundational pharmacokinetic and mechanistic insights, relevant preclinical studies were also included. Furthermore, to capture recent advances and ongoing research directions, we referred to clinical trial registration records (e.g., from ClinicalTrials.gov) with descriptions of the study designs, without extracting unpublished outcome data. The reference lists of retrieved articles were also screened to ensure the inclusion of seminal studies outside the primary search timeframe.

## 3 DME: pathogenesis and current management

### 3.1 Epidemiology

Diabetes mellitus (DM) is a chronic metabolic disorder affecting 537 million people globally in 2021 (9). Its most common microvascular complication—DR—affects up to 34.6% of diabetic patients worldwide (10), with a comparable prevalence of 34.08% in mainland China (11).

DME is the leading cause of vision loss in DR (12), occurring in 6.8% of diabetes patients aged 20–79 (13). Notably, disease duration correlates strongly with DME risk: no cases are reported within five years of DM diagnosis, while the incidence rises to 29% after 20 years (14).

### 3.2 Pathophysiology

A clear understanding of DME pathophysiology is essential for optimizing its treatment. Chronic hyperglycemia can induce retinal hypoxia, activating the hypoxia-inducible factor-1α (HIF-1α) signaling pathway and subsequently upregulating VEGF expression (15). Concurrently, it also stimulates the nuclear factor-kappa-B (NF-κB) pathway to promote proinflammatory cytokines (e.g., IL-6, TNF-α) release (16). Together, VEGF and inflammatory mediators phosphorylate tight junction proteins (e.g., occludin, claudins) in retinal microvascular endothelial cells, disrupting the inner blood-retinal barrier (iBRB) (17, 18). This disruption causes capillary fluid and protein leakage into the retina, forming extracellular edema (19). Since the macula is responsible for > 90% of central vision and color perception, macular edema can trigger vision loss, metamorphopsia, and reduced contrast sensitivity (20, 21). These impairments hinder daily activities like reading, driving, and are also associated with anxiety and depression, resulting in substantially reducing life quality (22).

**FIGURE 1:**
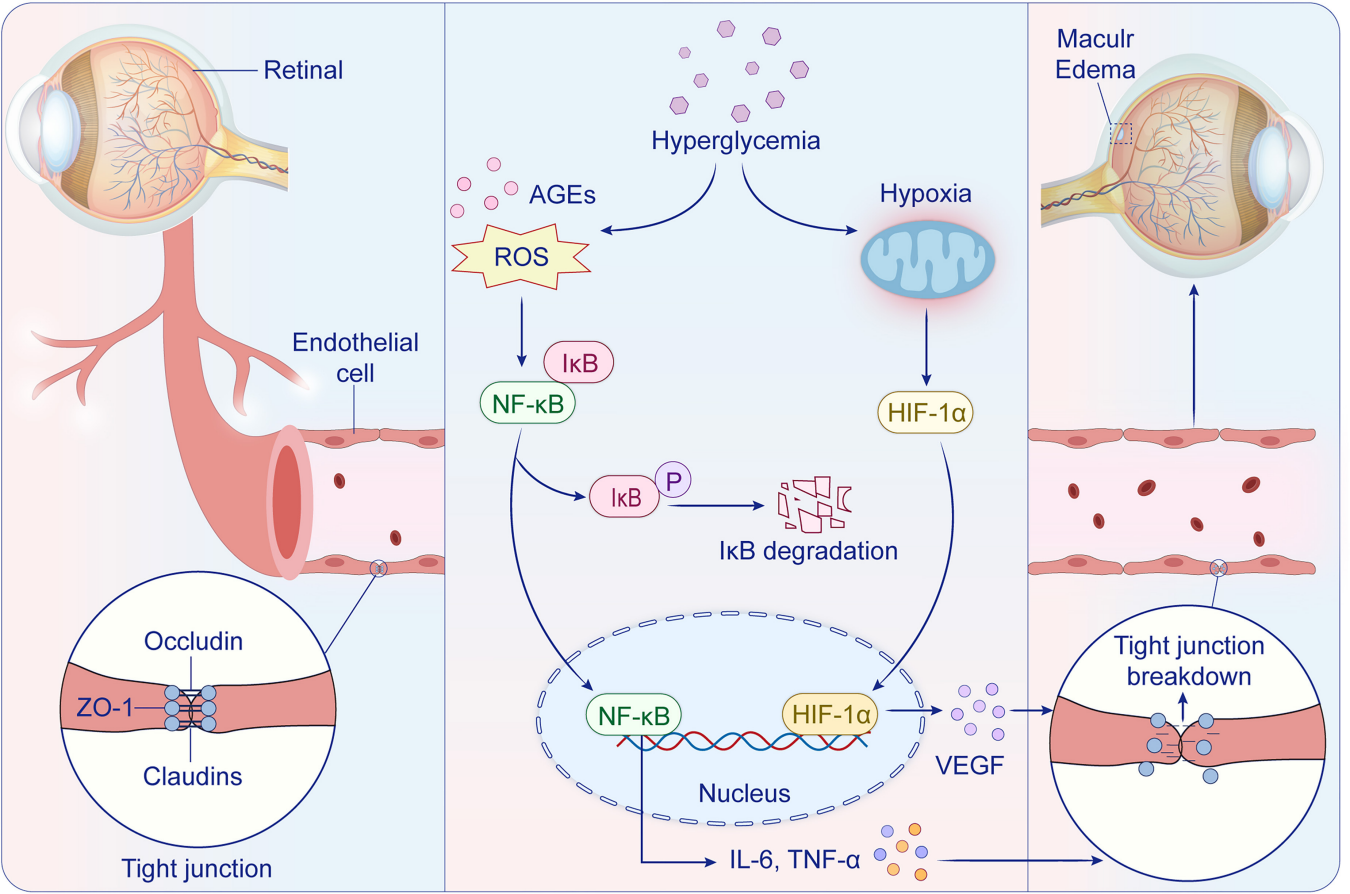
Pathogenesis of diabetic macular edema. Chronic hyperglycemia induces retinal hypoxia and AGEs/ROS, activating HIF-1α/NF-κB pathways. This upregulates VEGF and inflammatory cytokines, which disrupt the inner blood-retinal barrier, leading to increased vascular permeability and macular edema.

Therefore, timely intervention to resolve edema and preserve macular function is critical for diabetic patients (Figure 1).

### 3.3 Limitations of conventional therapies

Conventional laser photocoagulation has long been the gold standard for DME treatment. Its efficacy stems from occluding leaking microaneurysms and activating retinal pigment epithelial (RPE) cells to promote macular fluid resolution (23). However, irreversible neuroepithelial damage and high incidence of complications (e.g., macular puckering, visual field defects) (24–27) limit its long-term use.

Currently, IVI of anti-VEGF agents and sustained-release intravitreal corticosteroid implants are both established first-line therapies for DME. Anti-VEGF agents act via targeted VEGF, binding to block VEGF receptors interaction and reduce macular vascular leakage (28–30). Nevertheless, their efficacy is limited by several factors: (1) short drug half-lives necessitate frequent injections, lowering patient compliance; (2) repeated administration raises risks of endophthalmitis, elevated intraocular pressure (IOP), and retinal hemorrhage; (3) about 40% of patients show poor responses (31–33). These issues compound patient burden and strain healthcare resources (34).

As an alternative first-line approach, sustained-release intravitreal corticosteroid implants offer a distinct mechanism of action (35). They stabilize the blood-retinal barrier (BRB) by inhibiting leukostasis (36), and modulating VEGF signaling (37), thereby reducing vascular leakage. Pivotal trials, such as those by the DRCR Retina Network, have established the efficacy of these implants(35). However, long-term use of corticosteroids may increase the risk of ocular hypertension and cataracts, with cataract formation typically observed after multiple injections (e.g., following the third injection) rather than immediately after a single dose (38). These adverse effects limit their long-term clinical utility and highlight the need for safer alternatives.

## 4 SCS: anatomy and drug delivery rationale

### 4.1 Anatomical and biomechanical features

**FIGURE 2:**
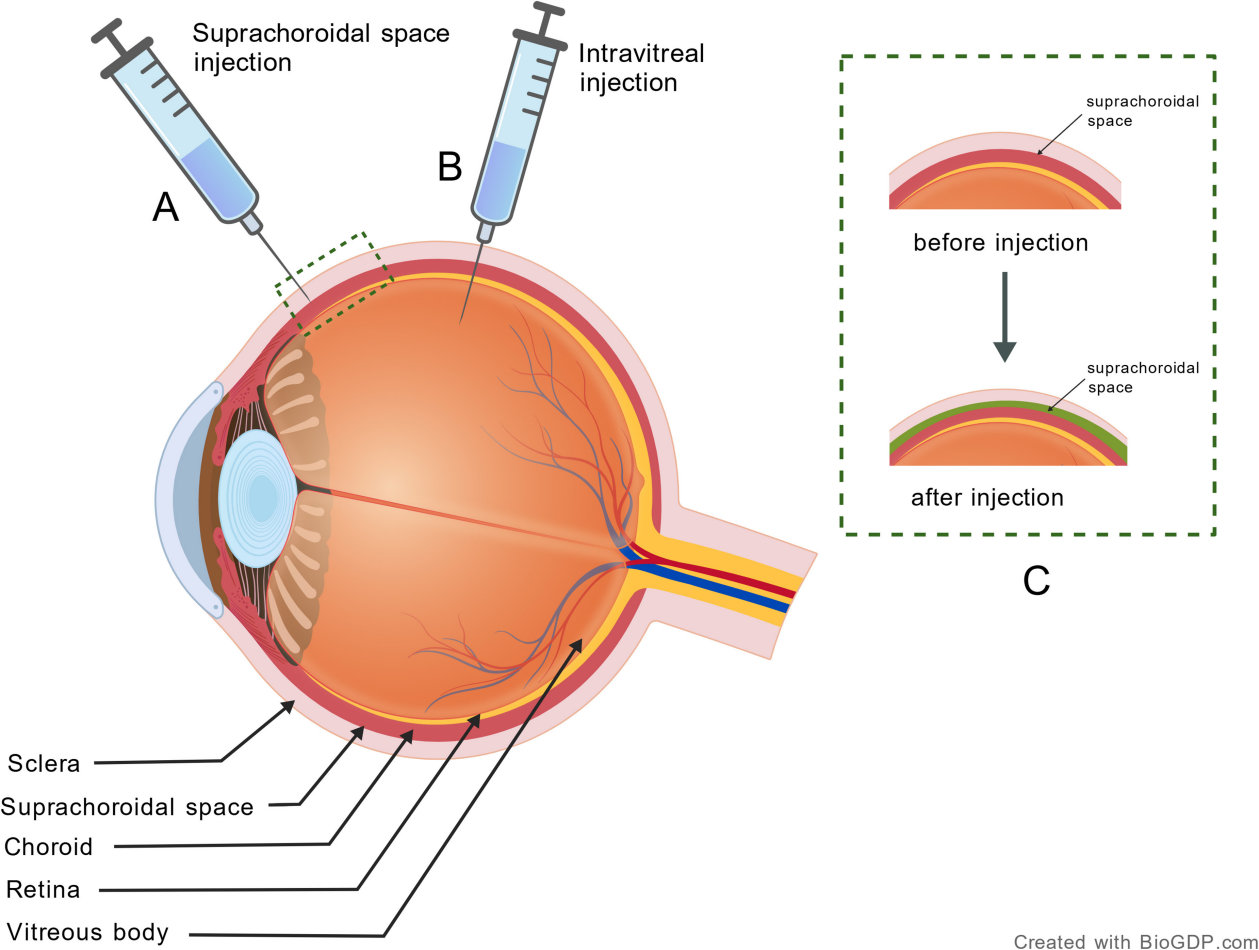
Anatomy and drug delivery routes of the suprachoroidal space. Schematic representation of drug delivery routes and tissue response: **(A)** suprachoroidal delivery route, **(B)** intravitreal injection route, and **(C)** resulting expansion of the suprachoroidal space after drug administration (green region). Created with BioGDP.com.

The SCS is a potential cavity located between the sclera and choroid, extending from the ciliary body anteriorly to the optic nerve posteriorly. Under normal conditions, it remains closely apposed due to IOP (39). The sclera comprises densely packed collagen fibers to form a rigid structure (40, 41), whereas the choroid is a vascular-rich and loose connective tissue with greater elasticity (42). Biomechanically, the sclera exhibits a compressive modulus approximately 7 times that of the choroid (43), allowing the choroid to deform more easily under pressure and facilitating SCS expansion. Moreover, the two layers are only connected by sparse fibrous tissue and scattered cells, lacking robust physical attachments (44), which further minimizes resistance during expansion. Therefore, these structural properties enable SCS expansion via fluid injection or mechanical cannulation, creating a potential reservoir for drug delivery (Figure 2).

Drugs injected into the SCS via the pars plana are naturally isolated from the anterior chamber, reducing drug exposure to anterior segment tissues. This minimizes drug effects on the lens and trabecular meshwork, lowering potential complications such as glaucoma and cataracts (45). Moreover, a physiological hydrostatic pressure gradient exists within the SCS: the pressure in the anterior SCS is approximately 0.8 ± 0.2 mmHg lower than IOP, while in the posterior SCS it is 3.7 ± 0.4 mmHg lower than IOP (46). This posteriorly decreasing pressure gradient (i.e., lower pressure in the posterior region relative to the anterior region) promotes fluid movement toward the posterior pole, enabling broad retinal contact and high local drug concentration. The injection-induced expansion can return to its pre-injection state after drug clearance (47), avoiding potential long-term detachment between the retina and choroid. While transient IOP elevation may occur post-injection, the peak value typically remains ≤ 30 mmHg and normalizes within 6–8 h, further supporting its safety (48).

### 4.2 Pharmacokinetics for posterior segment targeting

Pharmacokinetic studies demonstrated that SCS injection effectively targets drug delivery to the posterior eye.

In rabbit studies (49), injection of 50 μL TA via the SCS resulted in peak concentrations of 1,912 ng/mL in the posterior vitreous and 400,369 ng/g in the retina, while TA was below 1 ng/mL in aqueous humor, and 11.6 ng/mL at peak in plasma. Retinal exposure was 520,000-fold higher than that in the aqueous humor and 29,000-fold greater than that systemic exposure, highlighting exceptional posterior segment enrichment.

Furthermore, SCS injection achieved 10–100-fold higher drug concentrations in the chorioretinal region compared to that of IVI. For instance, TA exposure in posterior tissues (retina pigment epithelium, choroid, and sclera) increased 12-fold over that of IVI, while exposure in anterior tissues (lens, iris/ciliary body) and aqueous humor decreased by ≥ 96%, explaining the reduced risk of anterior segment complications (50).

### 4.3 Drug clearance and sustained-release strategies

Animal studies have shown that drugs administered via SCS are cleared more rapidly than those administered via IVI (51–53). For example, after SCS injection, bevacizumab became undetectable in ocular tissues within 7 days, whereas following IVI, it persisted in the retina, choroid, and vitreous for 30–60 days, with a gradual decline in tissue levels over time (53). Further investigations revealed that drug retention in the SCS is related to both formulation viscosity and drug particle size. Higher viscosity prolonged SCS closure: increasing viscosity from 75,000 ± 35,000 cPs to around 200,000 cPs extended closure time from 19 min to 9.2 days (54–56). Regarding particle size, both very small nanoparticles (e.g., 20 nm) and very large molecular complexes (up to 2 MDa) exhibited prolonged retention—up to 4 months and 21 days, respectively—compared with rapidly cleared small molecules. For example, micronized TA particles are retained in ocular tissues for at least 120 days, supporting their sustained-release profile and established use in DME treatment (57–59).

These pharmacokinetic properties underscore the potential of SCS drug delivery for targeted therapy with an improved safety profile, offering a rational basis for clinical optimization.

### 4.4 SCS drug delivery technologies

#### 4.4.1 Evolution of delivery systems

To achieve efficient drug delivery of SCS injection, various technical approaches have been developed.

Microcatheter insertion, an early exploratory method, involves a full-thickness scleral incision to guide a catheter into the SCS under light-emitting diode (LED) visualization. While it enables precise drug placement and real-time monitoring, it requires an operating room and may cause some complications (53, 60). Conventional hypodermic needles provide another alternative. Although cost-effective, this approach requires substantial operator skills to avoid adverse events such as scleral perforation and subretinal injection (61, 62). Recently, microneedle has emerged as an innovative approach for targeted SCS delivery. This hollow device, designed to slightly exceed sclera and conjunctiva thickness, incorporates a distal orifice for controlled drug release. Correct insertion is confirmed by a “loss of resistance” tactile feedback. Compared to conventional needles, microneedles exhibit advantages like easy operation, patient comfort, and simplified training (63, 64).

#### 4.4.2 Anatomical basis for microneedle design

The design of microneedle is guided by scleral anatomy, particularly its heterogeneous thickness. Human sclera varies regionally, measuring 800–1,000 μm at a point 2 mm posterior to the limbus, but only 300–500 μm near extraocular muscle attachment. The pars plana region, with a consistent thickness of 600–700 μm, offers an optimal insertion site due to its structural uniformity, which minimizes inter individual variation in required needle length and improves placement accuracy (65, 66).

Anterior segment optical coherence tomography (OCT) data indicate a mean human scleral thickness of 700–800 μm. With the conjunctiva adding about 200 μm, the total conjunctival–scleral thickness reaches approximately 900–1,000 μm. Consequently, an ideal SCS microneedle length is 900–1,000 μm (≈1 mm). This precise length ensures targeted delivery into the SCS while avoiding over-penetration and potential injury to deeper choroidal tissues. This design provides an anatomical foundation for safe and precise SCS access (67–69).

#### 4.4.3 FDA-approved drug-device combination

**FIGURE 3:**
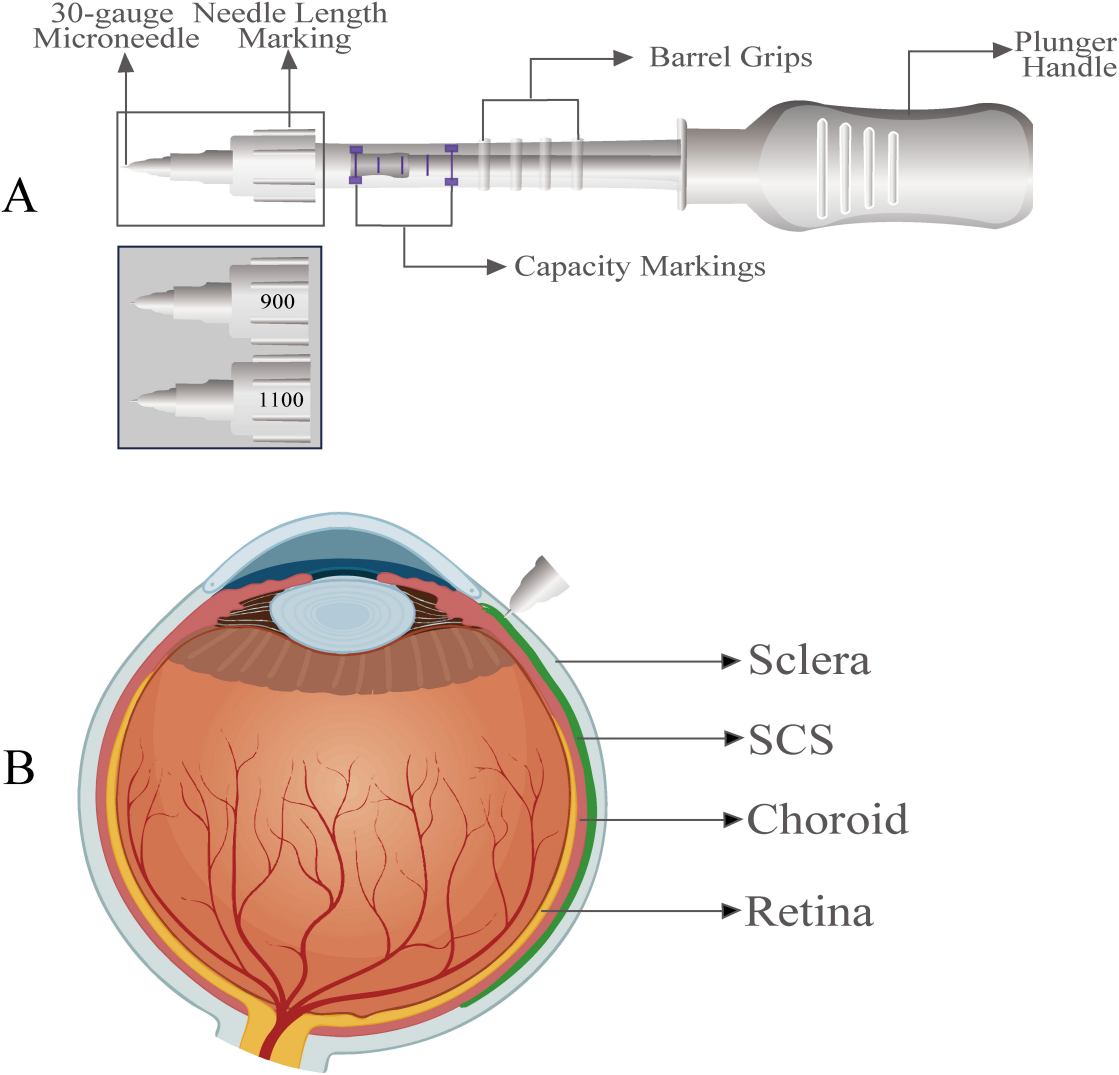
The SCS microinjector and its drug distribution pattern. **(A)** The SCS microinjector comprises a 30-gauge microneedle with length markings (e.g., 900 μm and 1100 μm), a conjunctiva-compressing hub, barrel grips, capacity markings, and a plunger handle for precise fluid delivery. **(B)** Schematic of drug distribution following suprachoroidal administration. The microneedle delivers therapeutics into the suprachoroidal space (SCS), with the green-highlighted area indicating circumferential drug distribution within this compartment (between the sclera and choroid).

The development of SCS drug delivery has achieved a major translational milestone with the FDA approval of Clearside Biomedical’s SCS Microinjector^®^ (Figure 3). As the first and only marketed SCS injection system, it establishes a key benchmark for clinical practice.

##### 4.4.3.1 Device characteristics

The core components of SCS Microinjector^®^ include a syringe handpiece for controlled injection force and an adapter-connected syringe for drug aspiration and storage. A replaceable microneedle assembly, available in s 30-G × 900 μm or 30-G × 1,100 μm stainless steel hollow microneedles, accommodates interpatient variations in scleral thickness. During injection, a conjunctival compression hub—an annular structure at the proximal end of the needle assembly—creates a fluid-tight seal by gently compression of the ocular surface. This key feature prevents reflux and leakage, thereby ensuring accurate administration of the agent into the target SCS region (63, 70, 71).

##### 4.4.3.2 Injection protocol

The SCS Microinjector^®^ procedure requires strict aseptic conditions. After ocular disinfection and local anesthesia, a 900 μm microneedle is inserted perpendicularly into the pars plana, 4.5 mm posterior to the limbus. Then a gentle compression is applied to the ocular surface to establish a conjunctival seal. The drug is injected steadily over 5–10 s while maintaining perpendicular needle orientation and compression. After injection, the needle remains in place for 3–5 s to facilitate initial drug dispersion. Withdrawal is followed by light pressure with a sterile swab for hemostasis. If resistance occurs, minor adjustments to the angle, compression, or injection site may be attempted. A 1,100 μm microneedle should be switched if there is a persistent resistance (70, 72).

## 5 Clinical trials of SCS injection for DME: efficacy and safety

To provide a clearer overview, the key clinical studies discussed below are summarized in Table 1.

**TABLE 1:** Summary of clinical trials evaluating SCS injection for DME.

Study/author (year)	Study design/phase	Sample size (eyes/patients)	Intervention		Key efficacy findings	Key safety findings and adverse events
HULK Trial Wykoff (73)	Phase 1/2, open-label, multicentre	20 patients (20 eyes) – Group 1:10 eyes (treatment-naive) – Group 2:10 eyes (Previously treated)	– Group 1: IVI of aflibercept + CLS-TA at baseline + PRN CLS-TA. – Group 2: CLS-TA monotherapy at baseline + PRN CLS-TA.	6 months	– BCVA: + 8.5 ETDRS letters (Group 1) vs. + 1.1 letters (Group 2)—CST: −91 μm (Group 1) vs. −128 μm (Group 2)	– Mean IOP: 13.8 → 14.2 mmHg—10% required IOP-lowering meds—3 cases of cataract progression—No injection-related serious AE
TYBEE Trial (74)	Phase 2, randomized, double-masked, parallel-design	71 patients (71 eyes)—Group 1: 36 eyes—Group 2: 35 eyes	– Group 1: CLS-TA + IVI of aflibercept at baseline and week 12 (+ sham IV at weeks 4, 8). – Group 2: IVI of aflibercept at baseline, weeks 4, 8, 12 (+ sham SCS at baseline, week 12). Both could receive additional aflibercept PRN from week 16	6 months	– BCVA: + 11.4 (Group 1) vs. + 13.8 letters (Group 2)—CST: −212.1 μm (Group 1) vs. −178.6 μm (Group 2)—Mean injections: 2.6 (Group 1) vs. 4.6 (Group 2)	– No treatment-related serious AEs—Ocular AEs (IOP elevation, cataract) comparable between groups—3 cases of IOP > 30 mmHg in group 1
Fazel et al. (75)	Phase 2/3, randomized, triple-blind	66 eyes (45 patients)—Group 1: 26 eyes—Group 2: 32 eyes	– Group 1: SCTA + 3 monthly IVB.—Group 2: Sham SCS + 3 monthly IVB.	3 months	– BCVA: Greater improvement in Group 1 (Δ -0.37 vs. −0.20 logMAR)—CST: −176 μm (Group 1) vs. −108 μm (Group 2)	– No significant IOP elevation or cataract progression. – Subconjunctival hemorrhage: 65.4% (Group 1) vs. 43.8% (Group 2), self-resolved.
Anwar et al. (76)	Prospective, observational, non-randomized	135 patients (135 eyes)—Group 1: 66 eyes (SCTA)—Group 2: 66 eyes (IVB)	– Group 1: Single SCTA. – Group 2: Single IVB	3 months	– Primary endpoint (≥ 5-letter BCVA gain + ≥ 10% CST reduction) at 3 mo: 37.5% (Group 1) vs. 29.5% (Group 2).—BCVA: + 5 letters (Group 1) vs. lower in Group 2—CST: ≥ 10% reduction more frequent in SCTA	– Comparable safety profiles between groups. – No detailed IOP or cataract data reported
Zakaria et al. (77)	Prospective, randomized comparative study	45 eyes (32 patients)—Group 1: 15 eyes (IVTA)—Group 2: 15 eyes (4 mg SCTA)—Group 3: 15 eyes (2 mg SCTA)	Single injection:—Group 1: 4 mg/0.1 mL—Group 2: 4 mg/0.4 mg SCTA 1 mL—Group 3: 2 mg/0.05 mL	6 months	– CST: Sustained reduction in Group 2 (–60.18 μm at 6 months); regression to baseline in Groups 1 and 3. -BCVA: Greatest improvement in Group 2 at 1 month.	– IOP elevation ≥ 10 mmHg: 1 (Group 1), 2 (Group 2), 1 (Group 3). – Cataract progression (phakic eyes): 30, 33.3, 25%, respectively. All managed medically
Shaikh et al. (78)	Quasi-experimental	4 patients (34 eyes)—Group 1: 17 eyes—Group 2: 17 eyes	– Group 1: Single IVTA (4 mg). – Group 2: Single SCTA (4 mg). Optional repeat dose at 6 weeks.	6 months	– BCVA and CMT: Comparable improvements at 3 months.—CST reduction maintained equally	– IOP at 6 months: Significantly higher in Group 1 (20.56 vs. 16.23 mmHg, *p* = 0.003). – Cataract progression faster in Group 1. – No serious acute AEs.
Marashi et al. (79)	Retrospective case series	11 eyes (10 patients)	– Single SCTA (4 mg/0.1 mL) using a custom-made needle.	8 weeks	– BCVA: Improved from 0.75 to 0.40 logMAR. – CMT: Reduced from 456.45 to 247.63 μm (45.7% reduction).	– Mean IOP stable (14.54 → 14.72 mmHg, *p* = 0.794). – No cataract progression. – No endophthalmitis/hemorrhage/detachment. – No other AEs reported

### 5.1 Initial studies: SCS injection as adjunct to anti-VEGF therapy

Early investigations into SCS injection for DME primarily explored its role as an adjunct to established anti-VEGF therapy, aiming to enhance efficacy or reduce treatment burden.

#### 5.1.1 HULK trial (NCT02949024)

20 DME patients were enrolled in the phase 1/2, open-label, multicenter HULK trial (73), categorized into treatment-naïve and previously treated arms. At baseline, the treatment-naïve eyes received a combination of suprachoroidal TA (CLS-TA, 0.1 mL/4.0 mg) with IVI of aflibercept (0.05 mL/2.0 mg), while the previously treated eyes received CLS-TA monotherapy. Both groups were followed by pro re nata (PRN) treatment. At 6 months, the treatment-naïve eyes showed greater best-corrected visual acuity (BCVA) improvement than that of previously treated eyes (+ 8.5 vs. + 1.1 ETDRS letters, p < 0.05). Anatomically, central subfield thickness (CST) decreased significantly in both arms (–128 μm in previously treated and –91 μm in treatment-naïve eyes). Safety assessments indicated a mild IOP increase from 13.8 to 14.2 mmHg in average, with only 10% patients requiring IOP-lowering medication. Cataract progression occurred in 3 patients, with no injection-related adverse events (AEs). While these results supported the potential of adjunctive CLS-TA, the study’s small sample size (*n* = 20), lack of a control group, and only 6-month follow-up significantly limit the strength of its conclusions, particularly regarding long-term safety effects such as sustained IOP elevation and cataract progression.

#### 5.1.2 TYPEE trial (NCT03126786)

This Phase 2 trial (74) evaluated a combination therapy of CLS-TA (4 mg) and IVI of aflibercept (2 mg) in 71 treatment-naïve DME patients, compared to IVI aflibercept monotherapy. While the combination group required fewer injections (2.6 vs. 4.6) and achieved a greater reduction in CST (–212.1 μm vs. –178.6 μm), the improvements in BCVA were comparable between groups (+ 11.4 vs. + 13.8 letters). The safety profile was favorable, with no occurrence of serious treatment-related AEs and ocular AEs that were comparable between two groups. These findings suggested that the combination regimen may reduce treatment burden and improve anatomical outcomes without compromising visual acuity. However, as a phase 2 trial, these findings require validation in larger, longer-term phase 3 trials to confirm durability of efficacy and long-term safety.

#### 5.1.3 Fazel et al. trial (IRCT20200314046761N1)

In this phase 2/3 trial (75), 66 patients with center-involved DME (CI-DME) were randomized to receive either a single SCS injection of TA(SCTA, 4 mg) plus 3 monthly IVI of bevacizumab (IVB; 1.25 mg), or a sham SCS injection plus 3 monthly IVB injections. At month 3, the combination group showed significant improvements in BCVA and reduction of CMT compared to that of the monotherapy group. Mean IOP remained stable, with no major AEs reported. Although these findings demonstrated the short-term benefits of SCTA as an adjunct to anti-VEGF therapy for the treatment of CI-DME, the pilot nature of the study and its brief follow-up duration restrict extrapolation to long-term efficacy and safety.

### 5.2 Comparative studies: SCS monotherapy vs. intravitreal therapy

Subsequent studies have directly compared SCS-TA monotherapy with conventional intravitreal injections to better define its relative efficacy and safety profile.

#### 5.2.1 SCTA vs. IVI Anti-VEGF

In a prospective study (76), 135 DME patients were enrolled and assigned to receive either IVB (2.5 mg/0.1 mL, *n* = 66) or SCTA (4 mg, *n* = 69). At a 3-month follow-up, more patients received SCTA met the primary endpoint of ≥ 5-letter BCVA gain plus ≥ 10% CST reduction than those received IVB. Both treatments demonstrated comparable safety profiles. This study suggested that SCTA may provide superior short-term visual and anatomical benefits over single IVB in the treatment of DME patients. However, the study’s short-term duration (3 months), lack of masking, and use of a non-standard, high-dose IVB (2.5 mg) limit the generalizability and interpretation of these findings.

#### 5.2.2 SCTA vs. intravitreal TA

A prospective RCT (77) assigned 45 DME eyes to receive an intravitreal TA (IVTA, *n* = 15; 4 mg/0.1 mL), 4 mg SCTA (n = 15; 4 mg/0.1 mL), or 2 mg SCTA (n = 15; 2 mg/0.05 mL). At month 3, only the 4.0 mg SCTA group maintained a significant central macular thickness (CMT) reduction (mean decrease of 16 μm from baseline), while CMT regressed to baseline in both the IVTA group and the 2.0 mg SCTA group. This suggests that superior durability of SCS-administered TA has been achieved at equivalent doses. All groups showed comparable complications regarding IOP elevation and cataract progression in short term. Notably, the 3-month follow-up represents a critical limitation, as corticosteroid-related complications such as IOP elevation and occurrence of cataract typically manifest over longer periods.

In a study of 34 refractory DME patients (78), participants received either a single IVTA (0.1 mL) or SCTA (0.1 mL), with optional repeat dosing at 6 weeks. During the 3-month assessment, BCVA improvements and CMT reduction were comparable at month 3. The IVTA group demonstrated significantly higher IOP level and faster cataract progression at month 3 and 6 compared with that in SCTA group. These observations indicated that SCS injection may offer long-term advantages in terms of control of IOP and cataract formation due to reduced anterior segment exposure, although the small sample size and variable retreatment protocol warrant cautious interpretation.

### 5.3 SCS injection in post-vitrectomy eyes

The utility of SCS injection has also been explored in challenging clinical scenarios, such as post-vitrectomy eyes where conventional drug clearance is altered. Marashi and Marashi (79) evaluated SCTA (4 mg/0.1 mL) in 11 post-vitrectomy DME eyes (1 phakic and 10 pseudophakic). At 8 weeks, significant improvements in BCVA and a 45.74% reduction in CMT were observed. No IOP elevations or cataract progression were noted in the phakic eye. These findings supported SCTA as an effective and well-tolerated treatment for post-vitrectomy DME patients. Given the very small sample size (*n* = 11), single-arm design, and short follow-up, these results are preliminary and require validation in larger studies.

## 6 Emerging technologies and future perspectives

Beyond the approved microinjector system, innovation in SCS drug delivery is advancing through both device optimization and novel therapeutic development, which pushes forward expanded clinical applications.

### 6.1 Device development

Multiple novel SCS devices are currently under clinical validation. A proprietary tissue separator was used to achieve tangential SCS access with the Everads Injector, potentially enabling improved drug distribution (80). A single 100 μL (4 mg) dose of TA was delivered with this minimally invasive device and an investigation is undertaken in an open-label pilot study (NCT06314217) assessing the safety and delivery performance among 10 previously treated DME patients (81).

Another development is the OXU-001 sustained-release dexamethasone formulation, designed for administration via the illuminated Oxulumis microcatheter system. An ongoing 52-week Phase II clinical trial (NCT05697809) is comparing two SCS-administered OXU-001 doses against the IVI dexamethasone implant (OZURDEX) for DME treatment (82–84). Positive outcomes could establish a minimally invasive approach providing sustained therapeutic effect for up to 12 months, potentially reducing treatment burden.

### 6.2 Sustained-release formulations

The development of long-acting formulations is advancing SCS therapy. Iluvien^®^, a non-biodegradable implant containing 0.19 mg of fluocinolone acetonide (FAc), provides sustained daily release of Fac (0.25 μg/day) for the treatment of chronic DME (85, 86). In a 12-patient retrospective study of chronic DME (87), the BCVA was improved (from 0.07 to 0.15 in decimal units) and CMT was reduced (from 544 to 404 μm) at month 12 with a single SCS implantation. In safety aspects, transient IOP elevation (< 10 mmHg, resolved within 3 weeks) was observed in 50% of patients, and mild nuclear sclerosis occurred in 20% of phakic eyes. No persistent ocular hypertension or surgery-related complications were reported.

### 6.3 Novel drug candidates

CLS-301, a pan-RGD integrin antagonist, can reduce vascular leakage and inflammation. In a study with rabbit (88), a single SCS injection (4 mg/eye) of CLS-301 suspension achieved sustained chorioretinal bioavailability over 16 weeks, with excellent ocular tolerability and no significant inflammation, supporting its potential as a long-acting DME therapy.

BCX4161, a plasma kallikrein inhibitor, was also evaluated following SCS administration (0.5 mg/eye) in rabbits. The inhibitor was well-tolerated over 3 months (89) (Muya et al. IOVS 2021; 62:ARVO E-Abstract 2194), with retinal drug concentrations exceeding the therapeutic target by 100–1,000-fold, demonstrating its potential for sustained and targeted DME therapy.

### 6.4 Nanocarriers and gene therapy

Explorations into nanocarriers and gene therapy are opening new avenues for long-term posterior segment treatment. Iron oxide/human serum albumin nanoparticles (IO/HSA NPs, 21 ± 3 nm) were efficiently delivered to the posterior segment. A single SCS injection in rats (90) (5 μL, 7.5 mg/mL) resulted in posterior segment retention for up to 30 weeks, with no significant retinal structural or functional changes, indicating both safety and potential for non-invasive tracking. RGX-314, an AAV8-based gene therapy encoding an anti-VEGF Fab, was evaluated in the phase II ALTITUDE trial (91) (NCT04567550) in DR patients. After SCS injection (2.5 × 10^11^ GC/eye) 33% of patients showed a ≥ 2-step DR improvement compared to that of control group at interim analysis, supporting the feasibility of SCS-mediated gene therapy.

## 7 Discussion

SCS injection presents a promising therapeutic strategy for DME, offering targeted posterior segment delivery while potentially reducing anterior segment complications. Preclinical pharmacokinetic studies have robustly demonstrated the targeted nature of this delivery method. Clinical evidence demonstrated that SCTA, alone or combined with anti-VEGF therapy, achieves both visual and anatomical improvements comparable to those of standard care, with a reduced treatment frequency and acceptable short-term safety.

The current evidence is predominantly from phase 1/2 studies, and the strength of evidence for efficacy and long-term safety remains low to moderate, and direct comparisons with the established standard of care, such as anti-VEGF monotherapy, are limited. Consequently, the precise role of SCS injection in the DME treatment algorithm remains to be defined. It may hold potential as an adjunctive therapy for patients with poor response to anti-VEGF agents.

The learning curve associated with the injection technique and the cost-effectiveness of this approach compared to existing therapies are also important considerations for future clinical adoption. Positioning SCS therapy within the context of established DME management will require further trials. With advancing technology and accumulating evidence, SCS delivery holds significant potential to become a cornerstone of effective, low-burden treatment for DME and other posterior segment diseases.
